# Species Diversity and Phylogeographical Affinities of the Branchiopoda (Crustacea) of Churchill, Manitoba, Canada

**DOI:** 10.1371/journal.pone.0018364

**Published:** 2011-05-17

**Authors:** Nicholas W. Jeffery, Manuel Elías-Gutiérrez, Sarah J. Adamowicz

**Affiliations:** 1 Department of Integrative Biology and Biodiversity Institute of Ontario, University of Guelph, Guelph, Ontario, Canada; 2 Department of Systematics and Aquatic Ecology, El Colegio de la Frontera Sur, Chetumal Unit, Quintana Roo, Mexico; University of Canterbury, New Zealand

## Abstract

The region of Churchill, Manitoba, contains a wide variety of habitats representative of both the boreal forest and arctic tundra and has been used as a model site for biodiversity studies for nearly seven decades within Canada. Much previous work has been done in Churchill to study the *Daphnia pulex* species complex in particular, but no study has completed a wide-scale survey on the crustacean species that inhabit Churchill's aquatic ecosystems using molecular markers. We have employed DNA barcoding to study the diversity of the Branchiopoda (Crustacea) in a wide variety of freshwater habitats and to determine the likely origins of the Churchill fauna following the last glaciation. The standard animal barcode marker (COI) was sequenced for 327 specimens, and a 3% divergence threshold was used to delineate potential species. We found 42 provisional and valid branchiopod species from this survey alone, including several cryptic lineages, in comparison with the 25 previously recorded from previous ecological works. Using published sequence data, we explored the phylogeographic affinities of Churchill's branchiopods, finding that the Churchill fauna apparently originated from all directions from multiple glacial refugia (including southern, Beringian, and high arctic regions). Overall, these microcrustaceans are very diverse in Churchill and contain multiple species complexes. The present study introduces among the first sequences for some understudied genera, for which further work is required to delineate species boundaries and develop a more complete understanding of branchiopod diversity over a larger spatial scale.

## Introduction

The sub-Arctic region of Churchill, Manitoba, Canada (58°46′N, 94°10′W) has been a model ecosystem for numerous biodiversity studies for nearly 70 years [1], [2]. Churchill represents the transition from the boreal forest to the arctic tundra and is located on the western shores of Hudson Bay. A wide variety of freshwater and marine habitats, including tundra ponds, quartzite rock bluff pools, lakes, streams, the Churchill River, and Hudson Bay itself, make Churchill an ideal location for sub-Arctic aquatic research. More recently, the freshwater environments of Churchill have been the focus of numerous studies on zooplankton population genetics [3]–[5], biogeography and phylogeography [6], [7], and community ecology [8], [9]. As this Churchill system is a model for aquatic biodiversity and ecology research, it is important to evaluate the species boundaries of the micro-invertebrates that live there. However, few studies have used DNA sequences to study the species diversity of microcrustaceans in this region, and no study has performed a multi-family assessment using sequences from a standardized gene region.

The majority of genetic studies that shed light upon species boundaries in the Churchill crustacean fauna have focused on the Cladocera, and, more specifically, the daphniids. The genus *Daphnia* is the best-studied group in Churchill; attempts to characterize the boundaries and members of the *Daphnia pulex* complex have evolved from using morphology, to allozyme comparison, to DNA sequencing. Weider and Hebert [4] used allozyme analysis of four loci to reveal multiple clonal “ecotypes” of *D. pulex* within the Churchill rock bluffs and tundra ponds. Later work by Colbourne et al. [10] used ND4 and ND5 mitochondrial sequences to reveal that the *D. pulex* complex is divided into nine distinct lineages (most of which likely correspond to species) in the Holarctic. Of these nine, only the *D. tenebrosa* lineage was represented among the individuals they sequenced from Churchill. Weider et al. [7] confirmed these results using restriction site polymorphisms in the ND4 and ND5 genes, and found that four additional lineages within the *D. pulex* complex alone are found in Churchill, including “panarctic *D. pulex*”, “western *D. pulicaria*”, “polar *D. pulicaria*”, and “*D. middendorffiana*”. These studies show that without genetic information the understanding of microcrustacean species diversity—and consequently their community associations—is problematic.

While many studies have used allozyme analysis and mtDNA sequencing to characterize individual species or species complexes of other branchiopod groups in North America, little research has been done to analyze the true diversity of the Branchiopoda in the Churchill region. For example, the water flea *Sida crystallina* consists of four distinct allopatric mitochondrial lineages within North America [11], but it is not known which of these inhabits Churchill. Similarly, several *Simocephalus* species [12] and *Polyphemus pediculus* [13] contain cryptic species diversity, but only a few samples from Churchill were included in these studies. Rowe et al. [14] showed that the morphospecies *Holpedium gibberum* in fact consists of two species: *H. gibberum* and *H. glacialis*. Although morphologically similar, these species possess important biological differences. While *H. glacialis* reproduces in the manner typical for cladocerans (cyclical parthenogenesis), *H. gibberum* within North America reproduces unisexually, either via selfing or obligate automictic parthenogenesis [15]. Since the Churchill region was not included in their studies, it is not known which *Holopedium* species (or both) inhabits this region, thus limiting our knowledge of community composition, biogeography, and the distributional limits of cyclic parthenogenesis in this genus.

The use of DNA sequences for studying species diversity is now a common practice and is particularly useful for taxa such as the microcrustaceans in which cryptic or nearly cryptic species diversity is very common. Occasionally, deeply divergent lineages that are not even sister taxa are contained within the same species name (e.g. *Daphnia pulex*, see [10]; *D. pulicaria* [16]). This confounds our understanding of species distributions, their environmental tolerances, and community associations and interactions. DNA barcoding—the use of short, standardized gene sequences for species identification and discovery—has become established as useful for validating species boundaries, conducting routine identifications, and revealing new and cryptic species [17]. Barcoding, which uses the mitochondrial gene cytochrome *c* oxidase subunit I (COI) for most animal groups, frequently reveals cryptic species diversity, for example within lepidopterans [18], amphipods [19], and also birds [20]. In freshwater zooplankton, barcoding recently allowed for the discovery and description of two cryptic species of cladocerans [21], [22] and one copepod [23]. It has been estimated that there may be approximately 2–4 times as many as the *c.* 1180 currently described species of branchiopod crustacean [24], [25], with unrecognized cryptic diversity expected to constitute a major portion of this undiscovered diversity.

This study uses DNA barcoding to investigate the species diversity of the Branchiopoda from Churchill, including both planktonic and benthic representatives from rock pools, tundra ponds, rivers, riparian backwaters, and lakes. For those genera that have been subjects of large-scale North American phylogeographic studies, we also determine which lineages inhabit the Churchill region. Further, we investigate the biogeographic origins of this fauna by examining the relative contributions of southern, Beringian, and high-Arctic elements to Churchill species composition. This study will contribute to our understanding of species boundaries and the true habitat tolerances of species, as well as having implications for the conservation of sub-Arctic freshwater fauna.

## Materials and Methods

### Animal husbandry and permit numbers

All animals collected were freshwater microcrustaceans and were immediately preserved in 95% ethanol in the field. This work was conducted under permits issued by the Manitoba Conservation Wildlife and Ecosystem Protection Branch from Winnipeg, MB to the Churchill Northern Studies Centre (CNSC) for conducting research in the Churchill Wildlife Management Area. The permit numbers are as follows: April 1 2008–March 31 2009 WB08445; April 1 2009–March 31 2010 WB09762. Prior to 2008, the same permits were issued to the CNSC but had no permit number associated with them.

### Specimen collection and identification

Sampling took place in July through the years of 2006–2009 and August 2006 from all types of inland aquatic habitats, including the Churchill River, various creeks, three large lakes, a wide variety of large and small tundra ponds, as well as the rock bluff pools near the shores of Hudson Bay. Collecting methods included both horizontal and vertical plankton tows using a 50 or 250 µm mesh plankton net, as well as free-hand sampling with dip nets from small ponds and littoral zones. Efforts were made to sample both benthic and pelagic species in areas with and without macrophytes present. Specimens were sorted to family, genus, or species when possible and were preserved in 95% ethanol which was changed once after 24 hours. Family and genus-level identifications were performed based on morphology [26], while most species-level identifications were done based on a combination of morphology and matching the Churchill sequences to published reference sequences. However, those without published DNA sequences were identified morphologically by the authors (see Table S1 in the supporting information online). It was possible to obtain identifications only to genus level for some specimens, some of which may represent new species.

Genetic clusters differing from one another by >3% COI divergence were considered provisional species for this study, as that level of divergence is often indicative of different species [17]. For provisional species lacking formal names, we used interim names according to existing codes used in the published literature whenever possible, when there was <2% genetic match to those records. This was done for the *Polyphemus* complex [13], the *Chydorus* complex [27], and *Sida crystallina* [11]. For lineages lacking genetic matches from the literature, we created interim names based on consecutive numbering of genetic clusters (those with >3% genetic divergence) within genera, combined with a designation of “NA” indicating the continent of North America (following an interim naming system introduced in Adamowicz et al. [28] for *Daphnia*). We aimed to sequence a sample size of at least 5 specimens per species when possible.

### DNA sequencing

Specimens were each photographed prior to DNA extraction. Typically, genomic DNA was extracted from entire specimens, but large specimens (>5 mm) were subsampled, with a piece of carapace or one or several limbs used. DNA was extracted and purified following the protocol of Ivanova et al. [29], using a 96-well filter plate with 1 ml wells and 3.0 µm glass fiber. The polymerase chain reaction (PCR) was used to amplify a ∼710 bp fragment of the 5′ region of the COI gene using the primer pair LCO1490 and HCO2198 [30], tailed with the M13 sequence [31]. The mini primers MLepF1 (Hebert, unpublished) and MLepR1 (Hajibabaei, unpublished) in combination with tailed Folmer primers, as well as crustacean-specific primers CrustF1 and CrustR1 [32], were attempted for some of the specimens that failed to amplify or sequence. The primers used for PCR and sequencing (along with all sequences, trace files, collection data, and photographs) are included for each specimen in the project Branchiopoda of Churchill [SABRA], available through the Barcode of Life Data Systems (BOLD) database [33].

PCR mixes had a total volume of 12.5 µl and contained 6.25 µl 10% trehalose, 2 µl ddH_2_O, 1.25 µl 10× Buffer for Platinum *Taq*, 0.625 µl of 50 mM MgCl_2_, 0.125 µl of each primer, 0.0625 µl 10 mM dNTPs, and 0.06 µl Platinum *Taq* polymerase. Each well contained 2 µl of DNA template. The thermocycling profile consisted of the COIfast reaction, which cycled each plate for one cycle of 1 min at 94°C; 5 cycles of 94°C for 1 min, 45°C for 40 sec, and 72° for 1 min; followed by 35 cycles of 94°C for 1 min, 51°C for 40 sec, and 72°C for 1 min; and then a final cycle of 72°C for 5 min. PCR products were electrophoresed in a 2.0% agarose E-gel (Invitrogen) stained with ethidium bromide and were visualized under UV light.

Each PCR product was sequenced bi-directionally on an ABI 3730XL DNA Analyzer (Applied Biosystems). A single consensus sequence was assembled using the forward and reverse sequences using CodonCode Aligner v. 3.0.2 (CodonCode Corporation).

### Knowledge of species diversity before vs. after genetic analysis

A checklist of species previously encountered at Churchill was compiled from the literature (Table S1 in the supporting information online). The type of habitat in which each species was found is also listed. For each genus, the previous species diversity was compared with the revised diversity following DNA barcoding. For the Chydoridae and Macrothricidae, family level was used due to uncertainty in some published identifications. The habitat(s) where each species was previously found were also compared to the habitat types from which we collected them.

### Analysis of genetic divergence

Using the sequence analysis tools available through BOLD [33], distance summary and nearest-neighbour (NN) analyses were performed for each species using the Kimura-2-Parameter (K2P) genetic distance model [34] with pairwise deletion of missing sites on all sequences >350 base pairs (bp). Mean intraspecific divergence, maximum intraspecific divergence, and mean NN distance (average distance to the nearest species) was calculated for each species, and the sample size was recorded. A neighbour-joining (NJ) phenogram was also constructed using the K2P distance model with a bootstrap test (1000 replicates) for all individuals with a COI sequence length >350 bp using MEGA version 4 [35]. Species clusters were collapsed using the Compress/Expand subtree function in the Tree Explorer module.

Provisional species differing by >5% are considered likely to be different species. In the Branchiopoda, when multiple types of evidence are available (ecological and/or nuclear markers to complement mitochondrial DNA), divergences of >5% are typically indicative of different species [24], [28]. Clusters showing 3–5% divergence were coloured red to reflect greater uncertainty of species status and the need for further study (e.g. involving morphology, ecology, or nuclear markers). While intraspecific variability is generally <2% [17], closely related species that have recently diverged can exhibit lower divergence; such cases were coloured blue, in a similar manner as a previous study of mammals in Asia [36].

### Phylogeographic analysis: a graphical approach

The distributions of the species or genetic clusters present in Churchill were investigated using a graphical approach using original and published COI data (available from GenBank or BOLD). Polygons were plotted on a map of North America showing the most distant points at which a particular genetic cluster (genetic divergences <2%) has been found to date. We used partially transparent polygons such that areas of overlap are darker; thus, the degree of shading on the map is proportional to the number of lineages likely colonizing Churchill from that region. This exploration of origins makes the assumption that most lineages will display continued occupancy of refugial regions combined with more recent expansion into glaciated regions, rather than large-scale shifts of entire ranges across major regions of the continent.

## Results

### Species encountered

In total, 42 species were collected, of which 41 were successfully sequenced (Table S1 in the supporting information online). Though we were able to collect several specimens of *Holopedium sp.* we were unable to obtain sequences for these specimens, and so they are not included in any genetic analyses. The overall sequencing success rate was 70% (327/466 specimens), but the high failure rate was mainly due to problematic genera including *Lynceus*, *Holopedium*, *Bosmina*, and *Scapholeberis*. Of the 139 specimens from which we were not able to obtain sequences at least 350 bp long, 72 of these belonged to these 4 genera alone. Multiple PCR attempts combined with using different primers resulted in success for 3 of these 4 genera. Of the 327 specimens for which we were able to obtain sequences, 294 of them were >500 bp had <1% undetermined nucleotides (Ns), and had two successful trace files, and were thus of barcode quality.

The family Daphniidae (order Anomopoda) was well represented at Churchill with 15 species encountered (Table S1 in the supporting information online). We were able to collect all 5 species of *Daphnia* previously recorded from the Churchill region—*D. magna* and 4 species within the *D. pulex* complex: *D.* cf. *pulex* sp. 2 NA (“panarctic *pulex” sensu* Colbourne et al. [10]), *D. pulicaria*, *D.* cf. *middendorffiana*, and *D. tenebrosa*. The *D. pulicaria* we encountered is likely the “polar *pulicaria*” mitochondrial lineage [10] as its closest genetic match was found in the high arctic. Three distinct genetic clusters were found within *Scapholeberis*, and one species of *Ceriodaphnia* was present. The genus *Simocephalus* was particularly diverse, with 6 potential species based on barcoding results.

The Chydoridae (Anomopoda) had the greatest diversity with 16 species, 5 of which were in the genus *Chydorus*. We also found multiple species within the genera *Eurycercus* (2 species), *Alona* (2), *Pleuroxus* (2), *Picripleuroxus* (2), and *Acroperus* (2), of which the latter 3 genera have not been reported from Churchill previously. The genus *Alonella* was represented by a single species among our samples.

Several other groups were present at much lower diversity. The families Bosminidae and Moinidae (Anomopoda) each contained just a single species, while the family Macrothricidae was represented by 2 species. The genus *Polyphemus* (order Onychopoda) contained 2 species, and the order Ctenopoda was represented only by *Sida* (1 species) and *Holopedium* (no sequences obtained). We collected three large branchiopod species, two anostracans (*Branchinecta paludosa* and *Eubranchipus bundyi*) and one laevicaudatan (*Lynceus* sp.). A notostracan (*Lepidurus arcticus*) has been previously recorded from Churchill, but we did not encounter this species.

DNA barcoding led to a marked increase in species diversity in some genera, as, for example, previously only one species of both *Chydorus* and *Simocephalus* had been reported from Churchill (Table S1 in the supporting information online).

### Patterns of genetic divergence

**Figure 1:**
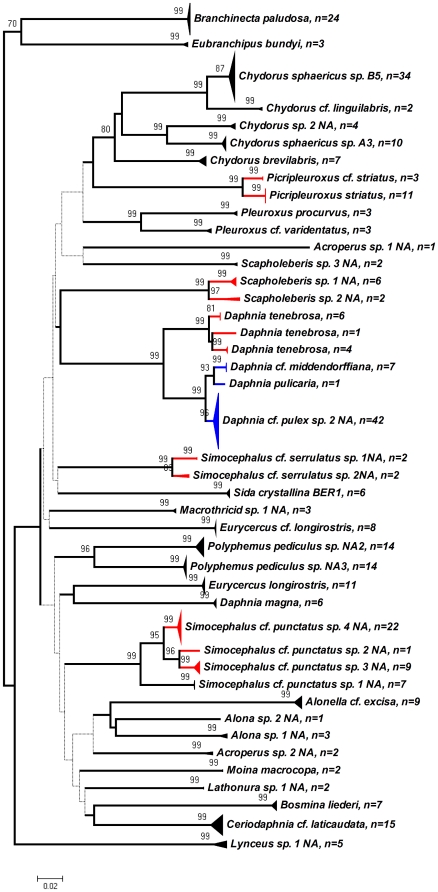
Neighbour-joining tree of COI sequences for all species of Branchiopoda obtained from Churchill, Manitoba. Species names and sample size are given for each branch. Red branches indicate genetic clusters (representing species or provisional species) separated from one another by a divergence of <5%, indicating that further study may be required to establish species status. *Daphnia tenebrosa* displays distinct intraspecific clusters, which differ from one another by an average of 2.5–3.1%, and are also coloured red. Blue colouring is used to highlight three species in the *Daphnia pulex* complex that each appear to form monophyletic mitochondrial lineages as indicated by bootstraps, but which show <2% COI divergence. The numbers above each branch indicate the level of bootstrap support (1000 replicates), and bootstrap values of less than 70% are indicated by the dotted lines.

After cutting the primer sites, the final sequences were 658 bp for those specimens successfully sequenced using Folmer primers. No indels or stop codons were observed in the alignment. The mean intraspecific divergence was 0.52%, while the maximum intraspecific divergence was 3.4% (range 0–3.4%). In most cases maximum intraspecific divergence was <2.4%, with the sole exception being found in *Daphnia tenebrosa*. By contrast, the average interspecific divergence between species within genera was 14.1% (range 1.87–32.3%) (Table 1). The mean distance to the nearest neighbouring species was 13.1% (range 1.9–27.4%). Almost all species displayed no overlap between the overall ranges of intraspecific and interspecific divergences (Table S2 in the supporting information online), with the exception of three members of the *Daphnia pulex* complex which were closely related (*D.* cf. *pulex* sp. 2 NA, *D. pulicaria*, *D.* cf. *middendorffiana*). Despite these low divergences, all branchiopod species studied displayed no overlap between their own intraspecific and interspecific divergences [37] and were supported by bootstrap values = 80% (Fig. 1).

**Table 1:** Overall COI distance summary at different taxonomic levels.

	No. of individuals	No. of taxa	Min. dist. (%)	Mean dist. (%)	Max. dist. (%)	SE dist. (%)
**Within species** 1	323	37	0	0.516	3.41	0.01
**Within genus**	327	20	1.87	14.1	32.3	0.14
**Within family**	327	10	19.1	26.3	36.6	0.029
**Within order**	327	5	18.9	27.5	40.4	0.023
**Within class**	327	1	19.9	29.7	43.1	0.031

1 Four species had only one sequence and so were not included in the within-species comparisons.

The minimum, mean (with standard error [SE]), and maximum pairwise COI sequence distance found among individuals within species, among individuals belonging to different species within genera, among genera within families, among families within orders, and among orders within the class for all specimens of Branchiopoda from Churchill analyzed in the present study that yielded sequences >350 bp.

Several morphospecies previously recorded from Churchill were found to have deeply divergent genetic clusters, reflecting the presence of cryptic diversity. For example, *Polyphemus pediculus* consisted of two clusters separated by a divergence of 15.2%. These corresponded to lineages NA2 and NA3 investigated by Xu et al. [13]. The morphospecies *Chydorus sphaericus* consisted of 2 distinct clusters that corresponded to lineages A3 and B5 found by Belyaeva and Taylor [27]; 3 additional species (*Chydorus brevilabris*, *C.* cf. *linguilabris*, and *Chydorus* sp. 2 NA) belonging to the *C. sphaericus* complex were also found in Churchill. In many additional cases, our results clearly matched the species found in Churchill with lineages investigated by other studies in North America that employed COI. For example, *Sida crystallina* lineage BER1 [11] was present. For some under-studied genera, there were no sequences available for comparison or no close matches.

Nine provisional species were separated from their nearest neighbours by 3–5% divergence, while 28 clusters were separated by >5% from all others. *Daphnia tenebrosa* was unique as it was represented by 3 distinct intraspecific clusters separated by averages of 2.5–3.1% divergence (total range of 0–3.4% divergence for 11 specimens in 3 clusters) (Fig. 1).

### Phylogeographic relationships and origins of the Churchill fauna

**Figure 2:**
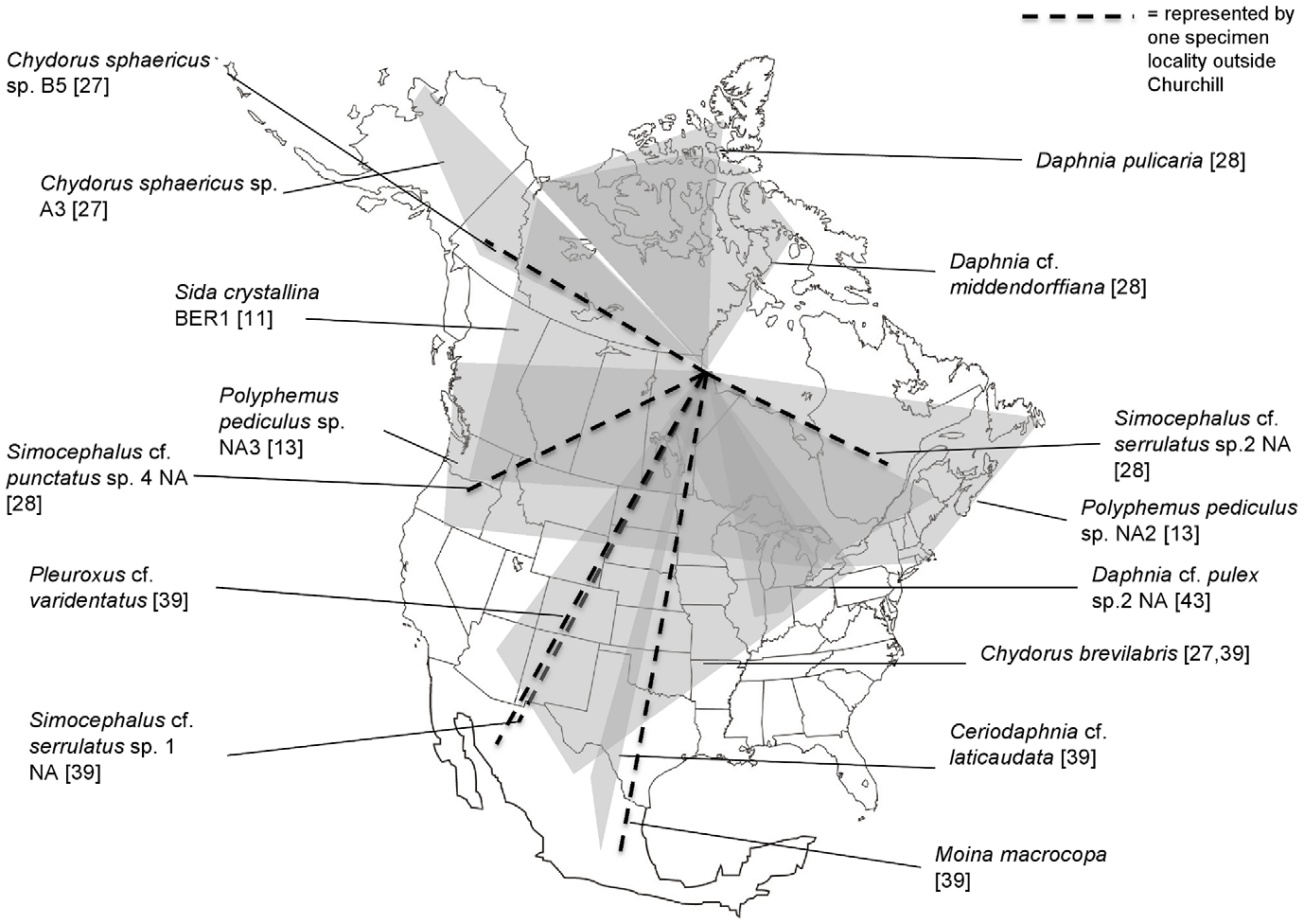
Map of North America showing the likely origins of the Churchill branchiopod fauna. The polygons represent distributions of species or genetic clusters with ≥98% COI similarity to the specimens we collected in Churchill. These also show likely colonization patterns of Churchill from various glacial refugia following the most recent glaciations. References for the matching sequences are provided in brackets.

Among the 41 species sequenced at Churchill, genetic matches (>98% similarity) were found from other regions for 15 of them. As seen in Fig. 2, the Churchill fauna appears to have originated from all directions, which correspond to the presence of multiple glacial refugia during the Pleistocene glaciations [6], [38]. Central and southern Mexico had the highest concentration of haplotype cluster ranges that were at least 98% similar in sequence to the Churchill fauna (5/15; *Simocephalus* cf. *serrulatus* sp.1 NA, *Moina macrocopa*, *Ceriodaphnia* cf. *laticaudata*, *Chydorus brevilabris*, and *Pleuroxus* cf. *varidentatus*), reflecting a southern origin. Other species originated from Beringia, including two members of the *Chydorus sphaericus* complex (*C. sphaericus* sp. A3 and sp. B5) as well as the Beringian *Sida crystallina* lineage. It is not clear whether *Daphnia pulicaria* and *D.* cf. *middendorffiana* colonized Churchill from refugia in the high arctic, or whether they colonized the high arctic from the south after the Pleistocene. The two divergent lineages of *Polymphemus pediculus* appear to have colonized Churchill from two separate regions, western and eastern temperate North America. Similarly, the two divergent lineages of *Simocephalus* cf. *serrulatus* appear to have colonized Churchill from two different regions, northwestern Mexico and eastern temperate North America.

## Discussion

The purpose of this study was to reveal the species diversity of Branchiopoda from the sub-Arctic region of Churchill, Manitoba, using DNA barcoding as a tool to help delineate provisional species boundaries. Our results show that the fauna in Churchill is much more diverse than previous checklists have shown, with an increase in the total known fauna from 25 to 42 described and provisional species collected during this study alone. We also demonstrated that by using standardized markers, it is possible to study species distributions, biogeographic patterns, and habitat occurrence, which is critical for community ecology studies. In particular, for some better-studied genera (e.g. *Daphnia*, *Chydorus*, *Polyphemus*, *Sida*), we were successful in matching our results to published sequences, thus creating a more complete understanding of diversity and distributions across a broader area. It appears that post-glacial colonization occurred from both the south and from the west (Beringia), and there may have been some species that arrived in Churchill from high-Arctic North American refugia or from polar Eurasian regions.

### Cryptic diversity in the Branchiopoda of Churchill

Cryptic diversity was revealed within several species and genera, including *Polyphemus pediculus*, *Simocephalus*, and several genera of Chydoridae. Cryptic diversity seems to be common among the Cladocera [39], and both *Scapholeberis duranguensis* and *Leberis chihuahensis* have been recently described after being highlighted by the barcode results from a study in Mexico. Establishing new species formally will take some time and could involve not only detailed morphological study, but ecophysiological characters and hybridization success rates as well [23]. This information will provide further understanding of local adaptation and microhabitat distribution in Churchill, and it will be especially important for understanding whether barcode clusters showing relatively shallow divergences (e.g. 2–5%) represent distinctive species or intraspecific phylogroups likely of glacial origin. Nevertheless, the barcode results prove useful to highlight provisional species, which will permit other types of analysis (biogeography and community associations) to be conducted in the immediate future, as these analyses would be much more time consuming if relying primarily on morphological data.

We found a particularly high level of local species diversity in the genus *Simocephalus* that builds upon previous findings of elevated species diversity when applying molecular tools to this group [12]. With only 3 species of *Simocephalus* previously known from North America, Hann [12] found 4 species complexes containing a total of 8 potential species for N. America using allozyme analysis. While Hann [12] found that *S.* cf. *vetulus* was the most widely distributed species and that it occurred in Churchill, our results indicated up to 6 putative species of *Simocephalus* in Churchill, none of which were identified as *S. vetulus*. Instead, our specimens appear to belong to the *S. punctatus* and *S. serrulatus* groups, the latter of which was previously thought to have a wide distribution in North America but relatively little intraspecific variation based on allozyme analysis. This suggests that more extensive sampling even within small regions, combined with employing DNA barcoding, increases our estimations of species richness in branchiopod groups.

### Standardization of identifications

Not only did our study split apart several morphospecies, but also several discrepancies from previous studies were revealed. We constructed a list consisting of 25 species from the literature, some of which are likely misidentified and are not actually found in Churchill.

Two species of *Ceriodaphnia* have previously been reported from Churchill from the rock bluffs alone, but after extensive sampling our barcode results show only one species, suggesting the possibility that previous identifications based on morphology in this genus may be incorrect. It is well known that this genus has several morphologies within the same species that can be confused [40]. Similarly, *Eurycercus lamellatus* was found by Shelford and Twomey [1], yet we identified the two lineages in Churchill as *E. longirostris* and *E.* cf. *longirostris*. While it is possible that these species were simply missed, after four years of extensive sampling we propose that this is unlikely.

Three main explanations can account for such discrepancies. The first possibility is misidentification by one or more studies. For example, although we sequenced 31 *Daphnia* specimens from rock bluff pools in our study, we did not detect *D. minnehaha* as reported by Ng et al. [9]. That record may have been a misidentification. Secondly, in other cases, we or others may have simply overlooked species which are rare or have cryptic life histories. Thirdly, in yet other cases, the discrepancies may reflect true changes in the species composition of the most common species over time, perhaps linked to climate change. However, drawing a solid conclusion about which of these 3 scenarios is responsible for a particular discrepancy is difficult to determine, due to a lack of vouchering in most ecological studies. It is not possible to determine if a previous author was in error, or whether we are, in the usage of a particular species name. For this reason, DNA barcoding is particularly useful for studying micro-invertebrate ecology and biodiversity. The DNA sequence data presented here will serve as a baseline survey which may be expanded. The sequences and vouchers will stay the same although the taxonomic names may be corrected or revised.

### Community associations of Churchill zooplankton: cases of cryptic cohabitation

It is possible that our results may influence and alter the results of previous community ecology studies that have taken place in Churchill. Hebert [8] noted that freshwater zooplankton communities are not particularly species rich, though it is likely that many communities are more so than previously thought. In many freshwater communities, the types of cohabiting species are limited through competitive interactions, but it is likely that there are cryptic species present in some cases, increasing diversity. This is particularly important for species conservation, as rare species may be missed due to the presence of other common zooplankton. Considering within-genus diversity only, many species seem to cohabit the same location and at the same time of year. For example, at the Churchill River weir site, on a single date both *Chydorus sphaericus* sp. A3 and B5 were found. Also at the Churchill weir and on a single date, *Simocephalus* cf. *punctatus* sp. 2, sp. 3, and sp. 4 were all found. Cases of co-habiting morphologically similar congenerics seem to be more common at complex sites with ample macrophytes and in benthic or littoral taxonomic groups. However, more research is needed to investigate levels of cohabiting diversity as well as species associations and exclusions, as we did not extensively sequence benthic specimens from single sites in this study.

There are also cases of multiple species within genera being found at the same site but at different dates. For example, at Goose Creek both *Polyphemus pediculus* sp. NA2 and sp. NA3 were found, but in different months. Also, at the same location, three species of *Chydorus* were encountered, again on different dates. Though this does not necessarily mean that these species do not co-occur in an area at the same time of year, it does show that the lack of understanding of cryptic diversity may result in overlooking cases of seasonal or inter-annual species turnover.

### The origin and structure of the Churchill biota

Overall our results suggest that the Churchill fauna originated from multiple glacial refugia (Fig. 2). Of the 15 species for which close matches were available among published sequences, it appears that at least 3 species originated from eastern temperate North America, 2 from western North America, 3 species from Beringia, 5 species from the southern central United States or Mexico, and 2 species possibly originated from high arctic refugia. It is possible that there were arctic refugia on the northern shores of Baffin Island, but it remains to be seen whether *Daphnia pulicaria* and *D.* cf. *middendorffiana* migrated to Churchill from this refuge or from another northern refuge in N. America or Eurasia. The majority of the species studied appear to have a boreal or temperate distribution, and only *D.* cf. *middendorffiana* [28], *D.* “polar *pulicaria*” [10], and *C. sphaericus* spp. A3 and B5 [27] have true arctic distributions. Although *Sida crystallina* BER1 appears to have originated from Beringia, this lineage also has a boreal distribution and so it is not a strictly arctic species, unlike the chydorids that originated from Beringia. It is important to note that our ability to discern these patterns is dependent upon published data, and it will be imperative to revisit these patterns once more extensive coverage for North America is available for all of the taxa in this study.

Hebert and Hann [6] found the highest diversity of freshwater crustaceans in low arctic sites, as well as in sites in Alaska that were found within the Beringian refuge. A decreasing diversity gradient was found when heading eastwards across the arctic. We have found that both eastward (from Beringia) and northward dispersal have been important for colonizing Churchill, with such mixing leading to a rich fauna. It is likely that species found in Churchill but not in high arctic sites are restricted in the north by a colder climate rather than limitations of dispersal. It is possible that there may not be any true decreasing gradient in total species richness while heading eastward across the arctic if molecular markers are used, as morphospecies may contain cryptic diversity from different source regions, and it will be useful to conduct more extensive surveys of Beringia and low arctic sites east of Beringia to study this pattern in more detail.

Hann [12] states that patterns of allozyme divergence in *Simocephalus* suggest the greatest amount of species differentiation occurred in the southeastern USA. While one of the species we encountered at Churchill (*S.* cf. *serrulatus* sp. 2 NA) matched to a sequence for a specimen found in the east coastal region, two other species were found on the west coast and the mid-west, suggesting that the southeastern USA may not have been the primary region of *Simocephalus* diversification in North America. However, there is still a lack of broad geographic coverage of molecular data for *Simocephalus*.

Some branchiopod species show very large distributions in North America. At least 7 of 61 species found in Mexico and Guatemala can be found in Churchill as well, but the majority of species have much narrower distributions [39]. Smaller-scale endemism is a common pattern amongst the Branchiopoda in North America, but further work is needed on the numerous understudied genera to investigate diversity and distributions on a global geographic scale.

### Conclusions

In conclusion, this study represents the first intensive DNA barcoding study on all groups of freshwater Branchiopoda from a sub-Arctic site. We found that the region of Churchill, Manitoba, contains higher microcrustacean diversity than previously recorded, and many cryptic, provisional species are likely to be present. While it is difficult to determine what species names should be applied to all genetic clusters, particularly in the absence of comparative data from Eurasia, these gene sequences represent valuable baseline knowledge about this fauna. Some clusters will gain names as comparisons are made, while others likely represent new species; nevertheless, the sequences will remain the same and serve as a record of the species present in Churchill during the timeframe of this study.

By matching COI sequences of some well-studied species, we were able to infer the phylogeographic origins of some of the Churchill fauna. It appears that Churchill was colonized from multiple directions following the Pleistocene glaciations, including the Beringian refuge, the southeastern and southwestern United States, as well as potentially a high arctic refugial area for *D.* cf. *middendorffiana* and *D. pulicaria*. While these results may simply reflect current species distributions, it is likely that most of these represent colonization events from those refugial regions due to the fact that some species (e.g. *Chydorus sphaericus sp. A3* and *sp. B5*) have only been found near specific refugia (in this case Beringia) despite broad study [27].

Future studies should focus on understudied groups for which there is currently little other molecular data. Many species were only given interim names due to the fact that many cryptic species have not been identified and described formally, and limited comparative data exist for many genera. It should also be a priority to formally describe species and to determine whether our shallower COI clusters represent species or glacial phylogroups using other life history, morphological, and ecological traits. Further genetic work on the Branchiopoda will help uncover their true diversity and distributions on a global scale. In particular, much more work is needed on the small-bodied, primarily benthic groups (such as Chydoridae) as our study revealed substantial diversity in this group. A comprehensive survey of DNA barcodes from different geographic regions will facilitate more objective research on the ecology, community associations, and environmental tolerances of aquatic biotas.

## Supporting Information

Table S1 **List of species found in Churchill from the literature versus the species revealed by DNA barcoding.** The results of our study show a large increase in the number of species compared to the number found in published literature on Churchill branchiopods. The type of habitat where each species was found is also listed. References are listed for both the original Churchill literature and for the studies from which we were able to obtain sequence matches of ≥98%. *Daphnia magna* and *D. tenebrosa* were identified morphologically and also had matches of ≥96% to published sequences.
(DOCX)

Table S2 **Summary of COI divergence patterns for all species with a sequence length >350 bp.** The mean and maximum intraspecific divergence found in all species which were successfully sequenced. The distance to the nearest neighbour is also provided, along with the sample size.
(DOCX)
