# Supplementary material for: Species Diversity and Phylogeographical Affinities of the Branchiopoda (Crustacea) of Churchill, Manitoba, Canada
Source: PLoS One. 2011 May 17;6(5):e18364. doi: 10.1371/journal.pone.0018364 (PMC3096620; doi:10.1371/journal.pone.0018364)
Supplement: Table S1 — List of species found in Churchill from the literature versus the species revealed by DNA barcoding. The results of our study show a large increase in the number of species compared to the number found in published literature on Churchill branchiopods. The type of habitat where each species was found is also listed. References are listed for both the original Churchill literature and for the studies from which we were able to obtain sequence matches of ≥98%. Daphnia magna and D. tenebrosa were identified morphologically and also had matches of ≥96% to published sequences. (DOCX) [file pone.0018364.s001.docx]

| **Species list and habitat occupancy from the literature** |  |  |  |  |  | **Species list and habitat occupancy based on the present study** |  |  |  |  |  |
| --- | --- | --- | --- | --- | --- | --- | --- | --- | --- | --- | --- |
|  | **Rock pools** | **Tundra ponds** | **Lakes** | **Rivers** | **References** | **Species** | **Rock pools** | **Tundra ponds** | **Lakes** | **Rivers/**  **backwater** | **References** |
|  |  |  |  |  |  |  |  |  |  |  |  |
| **Anomopoda** |  |  |  |  |  |  |  |  |  |  |  |
|  |  |  |  |  |  |  |  |  |  |  |  |
| **Bosminidae** |  |  |  |  |  |  |  |  |  |  |  |
|  |  |  |  |  |  | *Bosmina* liederi^1^ |  |  | X | X | Identified by SJA |
|  |  |  |  |  |  |  |  |  |  |  |  |
| **Chydoridae** |  |  |  |  |  |  |  |  |  |  |  |
|  |  |  |  |  |  | *Acroperus* sp. 1 NA |  |  |  | X | Identified by SJA |
|  |  |  |  |  |  | *Acroperus* sp. 2 NA |  | X |  | X | Identified by SJA |
| *Alona lapidicola* | X |  |  |  | [9] | *Alona* sp. 1 NA |  | X | X |  | Identified by SJA |
|  |  |  |  |  |  | *Alona* sp. 2 NA |  |  |  | X | Identified by MEG |
| *Alonella excisa* | X |  |  |  | [9] | *Alonella* cf. *excisa* |  | X |  |  | Identified by MEG |
| *Chydorus sphaericus* | X |  |  |  | [9] |  |  |  |  |  |  |
|  |  |  |  |  |  | *Chydorus brevilabris* |  |  | X | X | [27] |
|  |  |  |  |  |  | *Chydorus* cf. *linguilabris* | X |  |  |  | Identified by MEG |
|  |  |  |  |  |  | *Chydorus* sp. 2 NA |  | X |  | X | Identified by SJA |
|  |  |  |  |  |  | *Chydorus sphaericus* sp. A3 |  | X |  | X | [27] |
|  |  |  |  |  |  | *Chydorus sphaericus* sp. B5 | X | X |  | X | [27] |
| *Graptoleberis* sp. | X |  |  |  | [9] |  |  |  |  |  |  |
|  |  |  |  |  |  | *Picripleuroxus* cf. *striatus* |  | X |  |  | Identified by MEG |
|  |  |  |  |  |  | *Picripleuroxus striatus* |  | X |  |  | Identified by MEG |
|  |  |  |  |  |  | *Pleuroxus* cf. *varidentatus* | X |  |  | X | [32] for genetic match to *P. denticulatus*; morphological as *P. varidentatus* by MEG. Needs revision. |
|  |  |  |  |  |  | *Pleuroxus procurvus* |  | X |  | X | [27] |
| *Rhyncotalona* sp. | X |  |  |  | [9] |  |  |  |  |  |  |
|  |  |  |  |  |  |  |  |  |  |  |  |
| **Daphniidae** |  |  |  |  |  |  |  |  |  |  |  |
|  |  |  |  |  |  | *Ceriodaphnia* cf. *laticaudata* | X | X |  | X | [39] |
| *Ceriodaphnia pulchella* | X |  |  |  | [6,9] |  |  |  |  |  |  |
| *Ceriodaphnia reticulata* | X |  |  |  | [6,9,41] |  |  |  |  |  |  |
| *Daphnia magna* | X |  |  |  | [6,9] | *Daphnia magna* | X |  |  | X | 96% match [39] |
| *Daphnia middendorffiana* |  | X |  |  | [6] | *Daphnia* cf. *middendorffiana* | X |  |  |  | [28] |
| *Daphnia minnehaha^2^* | X |  |  |  | [9] |  |  |  |  |  |  |
| *Daphnia pulex* | X | X |  |  | [6,7] | *Daphnia* cf. *pulex* sp. 2 NA | X | X | X | X | [43] |
| *Daphnia pulicaria* | X |  |  |  | [7] | *Daphnia pulicaria* | X |  |  |  | [28] |
| *Daphnia tenebrosa* | X |  |  |  | [9,16] | *Daphnia tenebrosa* | X | X |  |  | 96% match [28] |
| *Moina macrocopa* |  |  |  |  | [39] | *Moina macrocopa* | X |  |  |  | [39] |
| *Moina rectirostris* | X |  |  |  | [6] |  |  |  |  |  |  |
|  |  |  |  |  |  | *Scapholeberis* sp. 1 NA |  |  |  | X^3^ | Identified by SJA |
|  |  |  |  |  |  | *Scapholeberis* sp. 2 NA |  |  |  | X^4^ | Identified by SJA |
|  |  |  |  |  |  | *Scapholeberis* sp. 3 NA |  |  |  | X | Identified by SJA |
|  |  |  |  |  |  | *Simocephalus* cf*. punctatus* sp.1 NA |  | X |  |  | Identified by MEG |
|  |  |  |  |  |  | *Simocephalus* cf. *punctatus* sp.2 NA |  |  |  | X | Identified by MEG |
|  |  |  |  |  |  | *Simocephalus* cf*. punctatus* sp.3 NA | X | X |  | X | Identified by MEG |
|  |  |  |  |  |  | *Simocephalus* cf*. punctatus* sp.4 NA | X | X |  | X | [28] |
|  |  |  |  |  |  | *Simocephalus* cf. *serrulatus* sp. 1 NA |  | X |  |  | [39] |
|  |  |  |  |  |  | *Simocephalus* cf. *serrulatus* sp. 2 NA |  |  | X |  | [28] |
| *Simocephalus vetulus* | X | X |  |  | [1,9,12,41] |  |  |  |  |  |  |
|  |  |  |  |  |  |  |  |  |  |  |  |
| **Eurycercidae** |  |  |  |  |  |  |  |  |  |  |  |
| *Eurycercus glacialis* |  | X |  |  | [41] | *Eurycercus* cf*. longirostris* |  | X |  |  | Identified by MEG |
| *Eurycercus lamellatus* |  | X |  |  | [1] | *Eurycercus longirostris* |  |  |  | X | 97% match [32] |
|  |  |  |  |  |  |  |  |  |  |  |  |
| **Macrothricidae** |  |  |  |  |  |  |  |  |  |  |  |
|  |  |  |  |  |  | *Lathonura* sp. 1 NA |  | X |  |  | Identified by SJA |
| *Macrothrix hirsuticornis* | X |  |  |  | [6] | Macrothricid sp. 1 NA |  |  |  | X | Identified by SJA |
|  |  |  |  |  |  |  |  |  |  |  |  |
| **Anostraca** |  |  |  |  |  |  |  |  |  |  |  |
|  |  |  |  |  |  |  |  |  |  |  |  |
| **Branchinectidae** |  |  |  |  |  |  |  |  |  |  |  |
| *Branchinecta paludosa* | X | X |  |  | [1,41] | *Branchinecta paludosa* | X | X |  | X | [44] |
|  |  |  |  |  |  |  |  |  |  |  |  |
| **Chirocephalidae** |  |  |  |  |  |  |  |  |  |  |  |
| *Eubranchipus bundyi* | X | X |  |  | [1] | *Eubranchipus bundyi* | X | X |  |  | [44] |
|  |  |  |  |  |  |  |  |  |  |  |  |
| **Ctenopoda** |  |  |  |  |  |  |  |  |  |  |  |
|  |  |  |  |  |  |  |  |  |  |  |  |
| **Holopedidae** |  |  |  |  |  |  |  |  |  |  |  |
|  |  |  |  |  |  | *Holopedium* sp.^5^ |  |  | X |  | Identified by SJA |
|  |  |  |  |  |  |  |  |  |  |  |  |
| **Sididae** |  |  |  |  |  |  |  |  |  |  |  |
| *Sida crystallina* |  |  |  |  | [42] | *Sida crystallina* BER1 |  | X |  |  | [11] |
|  |  |  |  |  |  |  |  |  |  |  |  |
| **Laevicaudata** |  |  |  |  |  |  |  |  |  |  |  |
|  |  |  |  |  |  |  |  |  |  |  |  |
| **Lynceidae** |  |  |  |  |  |  |  |  |  |  |  |
| *Lynceus brachyurus* |  | X |  |  | [42] | *Lynceus* sp. 1 NA |  | X |  |  | Identified by NWJ |
|  |  |  |  |  |  |  |  |  |  |  |  |
| **Onychopoda** |  |  |  |  |  |  |  |  |  |  |  |
|  |  |  |  |  |  |  |  |  |  |  |  |
| **Polyphemidae** |  |  |  |  |  |  |  |  |  |  |  |
| *Polyphemus pediculus* |  | X |  | X | [6,13] | *Polyphemus pediculus* sp. NA2 |  | X |  | X | [13] |
|  |  |  |  |  |  | *Polyphemus pediculus* sp. NA3 |  |  |  | X | [13] |
|  |  |  |  |  |  |  |  |  |  |  |  |
| **Notostraca** |  |  |  |  |  |  |  |  |  |  |  |
|  |  |  |  |  |  |  |  |  |  |  |  |
| **Triopsidae** |  |  |  |  |  |  |  |  |  |  |  |
| *Lepidurus arcticus* |  | X |  |  | [6] |  |  |  |  |  |  |

^1^ The genus *Bosmina* was found in multiple locations. While only short sequences of <350 bp were obtained from the lake specimens, the sequences indicated that the lake populations belonged to the same species that was present in the river marina.

^2^ Species may not be identified correctly.

^3^ Found in pool beside Churchill River.

^4^ Specimen found in pool beside Churchill River and also in a sample taken 4 km offshore in a Hudson Bay dredge, likely washed out to sea via the Churchill River.

^5^ *Holopedium* sp. localities are based on morphology only as no barcodes were obtained.

Additional references cited:

41. Boileau MG, Hebert PDN, Schwartz SS (1992) Non-equilibrium gene frequency divergence: persistent founder effects in natural populations. J Evol Biol 5: 25-39.

42. Beaton MJ (1988) Genome size variation in the Cladocera. *MSc Thesis*, University of Windsor

43. Paland S, Lynch M (2006) Transitions to asexuality result in excess amino acid substitutions. Science 311: 990-992.

44. Remigio EA, Hebert PDN (2000) Affinities among Anostracan (Crustacea: Branchiopoda) families inferred from phylogenetic analyses of multiple gene sequences. Mol Phylogenet Evol 17: 117-128.
