# Supplementary material for: Species Diversity and Phylogeographical Affinities of the Branchiopoda (Crustacea) of Churchill, Manitoba, Canada
Source: PLoS One. 2011 May 17;6(5):e18364. doi: 10.1371/journal.pone.0018364 (PMC3096620; doi:10.1371/journal.pone.0018364)
Supplement: Table S2 — Summary of COI divergence patterns for all species with a sequence length >350 bp. The mean and maximum intraspecific divergence found in all species which were successfully sequenced. The distance to the nearest neighbour is also provided, along with the sample size. (DOCX) [file pone.0018364.s002.docx]

| **Species** | **Mean within sp.** | **Max. within sp.** | **Distance to NN** | **Sample Size** |
| --- | --- | --- | --- | --- |
| **Order: Anomopoda** |  |  |  |  |
| **Family: Bosminidae** |  |  |  |  |
| *Bosmina liederi* | 0.71 | 1.46 | 24.29 | 7 |
| **Family: Chydoridae** |  |  |  |  |
| *Acroperus* sp. 1 NA | N/A | N/A | 27.35 | 1 |
| *Acroperus* sp. 2 NA | 1.13 | 1.13 | 19.79 | 2 |
| *Alona* sp. 1 NA | 0.63 | 0.98 | 16.67 | 3 |
| *Alona* sp. 2 NA | N/A | N/A | 16.67 | 1 |
| *Alonella* cf. *excisa* | 0.76 | 1.26 | 22.83 | 9 |
| *Chydorus brevilabris* | 0.77 | 1.3 | 14.12 | 7 |
| *Chydorus* cf. *linguilabris* | 0.49 | 0.49 | 5.45 | 2 |
| *Chydorus* sp. 2 NA | 0.8 | 1.43 | 9.2 | 4 |
| *Chydorus sphaericus* sp. A3 | 0.26 | 0.76 | 9.2 | 10 |
| *Chydorus sphaericus* sp. B5 | 0.72 | 2.17 | 5.45 | 34 |
| *Picripleuroxus* cf. *striatus* | 0 | 0 | 3.29 | 3 |
| *Picripleuroxus striatus* | 0 | 0 | 3.29 | 11 |
| *Pleuroxus* cf. *varidentatus* | 0.59 | 0.81 | 12.79 | 3 |
| *Pleuroxus procurvus* | 0.41 | 0.62 | 12.79 | 3 |
| **Family: Daphniidae** |  |  |  |  |
| *Ceriodaphnia* cf. *laticaudata* | 1.36 | 2.36 | 18.92 | 15 |
| *Daphnia* cf. *middendorffiana* | 0.09 | 0.33 | 1.88 | 7 |
| *Daphnia magna* | 0.28 | 0.49 | 21.05 | 6 |
| *Daphnia* cf. *pulex* sp. 2 NA | 0.45 | 1.41 | 1.87 | 42 |
| *Daphnia pulicaria* | N/A | N/A | 1.87 | 1 |
| *Daphnia tenebrosa* | 1.78 | 3.41 | 7.92 | 11 |
| *Moina macrocopa* | 0 | 0 | 19.33 | 2 |
| *Scapholeberis* sp. 1 NA  *Scapholeberis* sp. 2 NA | 0.57  2.42 | 1.06  2.42 | 3.85  3.85 | 6  2 |
| *Scapholeberis* sp. 3 NA | 0.43 | 0.43 | 21.1 | 2 |
| *Simocephalus* cf. *punctatus* sp. 1 NA | 0.07 | 0.18 | 7.2 | 7 |
| *Simocephalus* cf. *punctatus* sp. 2 NA | N/A | N/A | 3.18 | 1 |
| *Simocephalus* cf. *punctatus* sp. 3 NA | 0.32 | 0.96 | 3.18 | 9 |
| *Simocephalus* cf. *punctatus* sp. 4 NA | 0.29 | 0.77 | 3.31 | 22 |
| *Simocephalus* cf. *serrulatus* sp. 1 NA | 0 | 0 | 3.13 | 2 |
| *Simocephalus* cf. *serrulatus* sp. 2 NA | 1.86 | 1.86 | 3.13 | 2 |
| **Family: Eurycercidae** |  |  |  |  |
| *Eurycercus* cf. *longirostris* | 0.04 | 0.18 | 21.7 | 8 |
| *Eurycercus longirostris* | 0.24 | 0.61 | 21.28 | 11 |
| **Family: Macrothricidae** |  |  |  |  |
| *Lathonura* sp. 1 NA | 0 | 0 | 18.92 | 2 |
| Macrothricid sp. 1 NA | 0.41 | 0.46 | 19.53 | 3 |
| **Order: Anostraca** |  |  |  |  |
| **Family: Branchinectidae** |  |  |  |  |
| *Branchinecta paludosa* | 0.27 | 0.62 | 26.35 | 24 |
| **Family: Chirocephalidae** |  |  |  |  |
| *Eubranchipus bundyi* | 0.51 | 0.77 | 25.45 | 3 |
| **Order: Ctenopoda** |  |  |  |  |
| **Family: Sididae** |  |  |  |  |
| *Sida crystallina* BER1 | 0.2 | 0.54 | 22.4 | 6 |
| **Order: Laevicaudata** |  |  |  |  |
| **Family: Lynceidae** |  |  |  |  |
| *Lynceus* sp. 1 NA | 0.87 | 2.31 | 25.16 | 5 |
| **Order: Onychopoda** |  |  |  |  |
| **Family: Polyphemidae** |  |  |  |  |
| *Polyphemus pediculus* sp. NA2 | 0.75 | 1.55 | 15.15 | 14 |
| *Polyphemus pediculus* sp. NA3 | 0.12 | 0.55 | 15.15 | 14 |
